# P-1101. A Comparative Analysis of Resistance Rates Among *Escherichia coli* and *Klebsiella pneumoniae* Isolates Collected from the Gepotidacin Uropathogen Global Surveillance Studies and Phase 3 Uncomplicated Urinary Tract Infection Clinical Studies

**DOI:** 10.1093/ofid/ofae631.1289

**Published:** 2025-01-29

**Authors:** Renuka Kapoor, Deborah Butler, John Breton, Cara Kasapidis, Derrek Brown, Amanda Sheets, Didem Torumkuney, S J Ryan Arends, Rodrigo E Mendes, Nicole E Scangarella-Oman

**Affiliations:** GSK, Atlanta, Georgia; GSK, Atlanta, Georgia; GSK, Atlanta, Georgia; GSK, Atlanta, Georgia; GSK, Atlanta, Georgia; GSK, Collegeville, PA, USA, Collegeville, Pennsylvania; GSK, Atlanta, Georgia; Element, Iowa City (JMI Laboratories), North Liberty, Iowa; Element, Iowa City (JMI Laboratories), North Liberty, Iowa; GlaxoSmithKline plc., Collegeville, Pennsylvania

## Abstract

**Background:**

Gepotidacin (GEP) is a novel oral antibacterial under development for the treatment of uncomplicated urinary tract infection (uUTI) and gonorrhea. This study reports drug resistance rates for large collections of *Escherichia coli* (EC) and *Klebsiella pneumoniae* (KPN) isolates from the GEP uropathogen global surveillance studies vs isolates from 2 global GEP Phase 3 (Ph3) uUTI trials.

**Figure:**
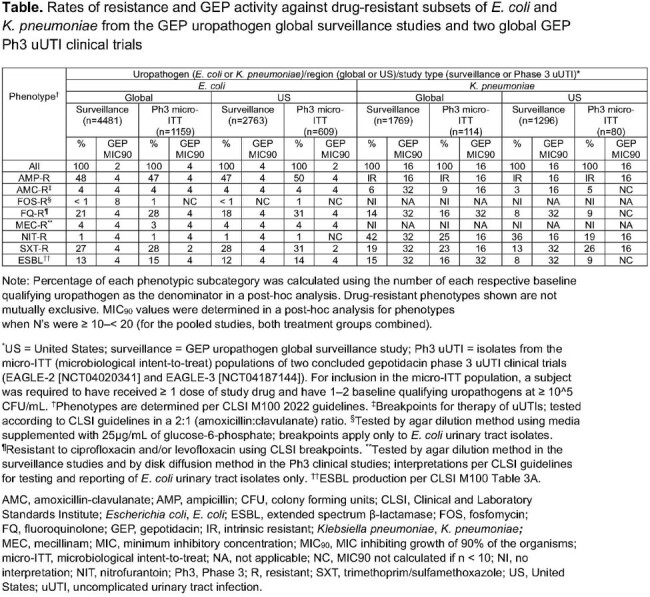

**Methods:**

A total of (G [global]/US [a subset of global isolates from the United States]) 4481/2763 EC and 1769/1296 KPN isolates were collected from the GEP uropathogen global surveillance studies (2019–2022). From an unbiased collection of isolates from all enrolled participants with uUTI, a total of (G/US) 1159/609 EC and 114/80 KPN isolates were from the microbiological intent-to-treat population in the GEP uUTI Ph3 clinical studies. All isolates in both studies were cultured from urine collected from patients in outpatient settings. Isolate identification was confirmed by MALDI-TOF and susceptibility testing by CLSI methods was conducted at a central laboratory (JMI Laboratories [surveillance] and PPD GCL [clinical studies]). MICs for oral antibiotics used for the treatment of uUTI and drug-resistant subsets were interpreted per CLSI guidelines.

**Figure:**
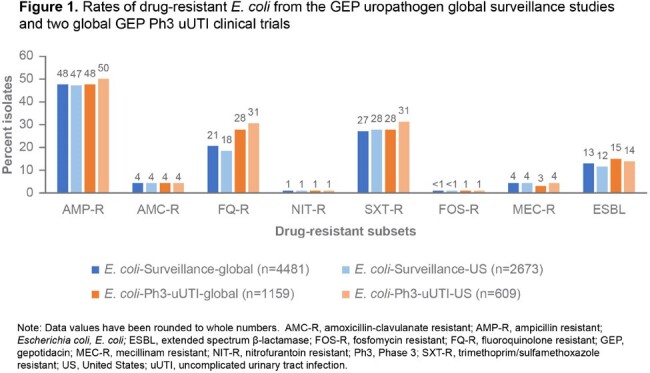

**Results:**

**EC**: FQ-R rates were 18% and 31% for the US and 21% and 28% for G isolates from the surveillance and Ph3 studies respectively. Resistance rates for SXT and AMP were ≥ 27%; rates for AMC, NIT, FOS, and MEC were ≤ 4% for both studies. ESBL+ % range was 12–15% in both studies (Figure 1).

**KPN**: Rates of FQ-R were similar for US (8%, 9%) and G isolates (14%,16%) from the surveillance and Ph3 studies respectively. Resistance rates for other antibacterials were 19–42% (NIT-R), 13–26% (SXT-R), and 8–16% (ESBL+ %) (Figure 2).

**Figure:**
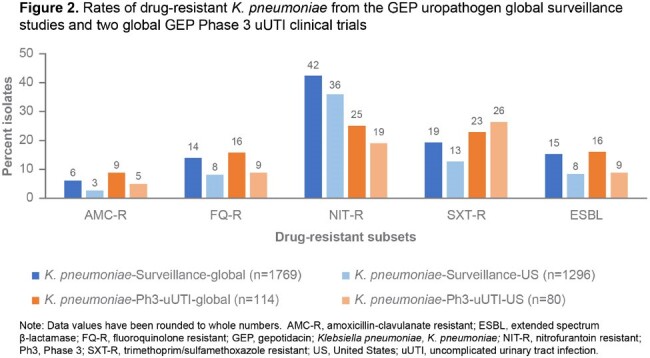

**Conclusion:**

Drug resistance rates to many uUTI antibacterials tested were high in 1 or both studies for EC and KPN. GEP MIC90s were generally the same or 1 dilution higher than all isolates for resistant subsets of EC and KPN among both surveillance and Ph3 isolates (Table).

**Funding:**Surveillance studies and EAGLE-2 were funded in part by GSK and in part with Federal funds from the US Office of the Assistant Secretary for Preparedness and Response, Biomedical Advanced Research and Development Authority (HHSO100201300011C). EAGLE-3 was funded by GSK.

**Disclosures:**

**Renuka Kapoor, PhD**, GSK: Employee|GSK: Stocks/Bonds (Public Company) **Deborah Butler, PharmD**, GSK: Employee|GSK: Stocks/Bonds (Public Company) **John Breton, MCM**, GSK: Employee|GSK: Stocks/Bonds (Public Company) **Cara Kasapidis, BS**, GSK: Employee|GSK: Stocks/Bonds (Public Company) **Derrek Brown, BS**, GSK: A former agency worker at GSK **Amanda Sheets, PhD**, GSK: Employee|GSK: Stocks/Bonds (Public Company) **Didem Torumkuney, PhD**, GSK: Employee|GSK: Stocks/Bonds (Public Company) **S.J. Ryan Arends, PhD**, JMI: SJRA is an employee of JMI. JMI was contracted by and received financial support from GSK to conduct gepotid **Rodrigo E. Mendes, PhD**, JMI: RM is an employee of JMI. JMI was contracted by and received financial support from GSK to conduct gepotidac|Paratek Pharmaceuticals: Advisor/Consultant|Paratek Pharmaceuticals: Grant/Research Support **Nicole E. Scangarella-Oman, MS**, GSK: Employee|GSK: Stocks/Bonds (Public Company)

